# Health Demands Moderate the Link Between Willpower Beliefs and Physical Activity in Patients with Knee Osteoarthritis

**DOI:** 10.1007/s12529-020-09865-w

**Published:** 2020-03-11

**Authors:** Sally Di Maio, Jan Keller, Veronika Job, Dieter Felsenberg, Wolfgang Ertel, Ralf Schwarzer, Nina Knoll

**Affiliations:** 1 Department of Education and Psychology, Health Psychology, Freie Universität Berlin, Habelschwerdter Allee 45, D-14195 Berlin, Germany; 2 Faculty of Psychology, Technische Universität Dresden, Dresden, Germany; 3 Center for Muscle and Bone Research, Universitätsmedizin Berlin, Berlin, Germany; 4 Department of Traumatology and Reconstructive Surgery, Universitätsmedizin Berlin, Berlin, Germany; 5 SWPS University of Social Sciences and Humanities, Wroclaw, Poland

**Keywords:** Osteoarthritis, Self-control, Willpower beliefs, Physical activity, Depressive symptoms, Accelerometer

## Abstract

**Background:**

Regular physical activity (PA) was found to alleviate pain and improve functioning among patients with osteoarthritis of the knee (OAK). Heightened health demands due to OAK severity, body mass index (BMI), and depressive symptoms may require self-regulatory strategies to engage in more PA. Research on willpower—the capacity to exert self-control—suggests that believing that willpower is a nonlimited rather than a limited resource predicts effective self-regulation specifically when demands are high. The present study examines the association of OAK patients’ willpower beliefs with their daily PA as a function of health demands.

**Methods:**

To identify the moderating role of OAK severity (WOMAC), BMI, and depressive symptoms (CES-D) on the link between willpower beliefs and objectively assessed PA over a 7-day period, baseline data of a registered randomized controlled trial with 243 patients (*M*_age_ = 65.47 years, *SD* = 0.49) were examined in secondary analyses.

**Results:**

Moderation analyses revealed that overall positive associations of willpower beliefs with PA were further qualified by OAK severity, BMI, and depressive symptoms. When patients faced less health demands, believing that willpower is nonlimited was associated with more PA. When health demands were higher, willpower beliefs were not associated with PA.

**Conclusion:**

OAK patients’ willpower beliefs were associated with PA. However, facing more health demands seemed to erase this beneficial link. Improving willpower beliefs by way of intervention may help to shed more light on predictive direction and ways to overcome barriers to regular physical activity.

## Introduction

Osteoarthritis of the knee (OAK) represents a major public health issue contributing to disability and increasing functional loss [1]. To reduce pain and improve functional health, patients with OAK are advised to engage in regular physical activity [2]. Despite its importance, low physical activity is prominent especially in adults with OAK [3]. Many individuals with OAK are challenged by a number of health demands that deter them from becoming physically active. Specifically, OAK symptom severity resulting in disability due to pain and stiffness of the joints, body mass index (BMI), and depressive symptoms have been shown to be main barriers of physical activity in this population [4, 5]. Not surprisingly, the insidious relationship between health demands and physical activity bears the risk for OAK patients to fall into a vicious cycle. Given low physical activity, OAK severity, including pain and stiffness, is more likely to aggravate and then challenge or even further diminish the capability to become physically active [4]. Additionally, there is a high risk for overweight or obese adults to develop OAK over their life course [6]. Making matters worse, overweight and obese adults with OAK report greater self-reported disability and engage in less physical activity than their normal-weight counterparts [4]. Not only burdened by physical strain, patients with OAK bear a two-fold risk of experiencing depressive symptoms [7]. In sum, regarding engagement in physical activity, patients with OAK face heightened self-regulatory demands due to potentially numerous challenging health demands. To cope with these demands, they need *self-control*, the capacity to advance distal goals over competing short-term motivation [8]. That is, to promote their long-term health goals by engaging in physical activity; patients with OAK often have to incur short-term adversities, such as pain, and overcome the immediate needs and habits of inaction.

A growing body of research indicates that individual differences in self-control performance depend on people’s beliefs about the availability of self-control resources, colloquially called *willpower* [9, 10]. According to this research, people differ in whether they believe that willpower is easily depleted and needs to be refueled after a demanding task (i.e., believing in limited willpower) or that willpower is rather nonlimited resource (i.e., believing in nonlimited willpower) that is not easily used up or can even be replenished when people exert self-control [11].

Believing that willpower is nonlimited may crucially enable the capacity to exert self-control especially when self-control demands are high [9, 11–14]. For instance, beliefs in nonlimited willpower were functional for sustained performance in multiple consecutive mental self-control tasks [11, 13] and attenuated the need for recovery most notably after a strenuous day at work [14]. With reference to health-related behaviors, beliefs in nonlimited willpower were linked to less unhealthy eating only during times of heavy course load [11, 12] and better management of type 2 diabetes and less diabetes-specific distress particularly among newly diagnosed patients (i.e., circumstances with high self-control demands) versus those familiar with the diagnosis [9].

Two different mechanisms have been proposed to explain the effects of willpower beliefs on self-control performance. On one hand, it has been shown that having exerted self-control was associated with reduced self-efficacy in people with a limited theory about willpower [15]. This temporary reduction in self-efficacy may, in turn, impair their further performance. On the other hand, motivational shifts following self-control exertion may explain the effects of willpower beliefs. When they have exerted effort and potentially feel exhausted, people who believe that willpower is limited experience a need to recover and replenish their resources and accordingly, prefer passive, resting activities. Such a motivational shift is typically not observed in people who think that willpower is not limited [16].

To sum up, according to willpower theory, individuals’ beliefs in willpower as a limited resource contribute to self-regulation failure [11]. Those believing in nonlimited willpower are assumed to be especially capable to self-control when the necessity to repeatedly exert self-control is high.

## Aims and Hypotheses

As reviewed above, OAK severity, including pain and stiffness; BMI; and depressive symptoms produce heightened self-regulatory demands regarding the engagement in regular physical activity. As a function of such varying health demands (i.e., OAK severity, BMI, depressive symptoms), the relationships of patients’ willpower beliefs with their daily moderate-to-vigorous physical activity (MVPA) and their daily steps are examined in the present study. With reference to the above reviewed propositions and findings from willpower theory [9, 11–14], we expect that patients reporting beliefs in nonlimited willpower will more likely show higher daily MVPA and steps than those reporting beliefs in limited willpower (hypothesis 1). Moreover, we assume that positive links between willpower beliefs and physical activity outcomes are moderated by health demands. That is, we expect nonlimited willpower beliefs combined with more severe health demands (i.e., *M* + 1 *SD*; OAK severity, BMI, or depressive symptoms) to be associated with higher physical activity levels than when combined with less severe health demands (i.e., *M* − 1 *SD*; hypothesis 2).

## Methods

### Participants

This study includes secondary analyses on baseline data of a 2-year pre-registered randomized controlled trial (registration number: DRKS00009677; German Clinical Trials Register) designed to facilitate physical activity in adults with OAK. Eligibility criteria, study design, and recruitment strategies of the trial are reported in the study protocol by Knoll et al. [17]. The final sample encompassed *N* = 243 patients with OAK of which *n* = 152 (62.6%) were women. Patients’ mean age was 65.47 years (*SD* = 0.49) ranging from 44 to 80 years. More than half of the sample were retired (*n* = 141, 58.0%), *n* = 84 (34%) patients reported to be employed, and *n* = 18 (7.4%) reported to be unemployed. Most patients were married or reported to live in a relationship (*n* = 159; 65.4%), *n* = 26 (10.7%) patients reported not living in a relationship, *n* = 43 (17.7%) were divorced, and *n* = 20 (8.2%) widowed. The majority of patients had children (*n* = 182; 74.9%). Approximately half of the sample reported having a university degree (*n* = 115; 47.3%).

### Procedure

After patients received detailed study information and provided written informed consent; research staff assessed weight and height using objective measures. Subsequently, patients completed questionnaires which included items on socio-demographic variables; willpower beliefs; OAK severity, including pain and stiffness; and depressive symptoms. Trained research personnel provided instructions on wearing the accelerometer devices. Patients were asked to wear the devices strapped around their hips for the following week throughout waking hours, apart from water activities.

### Measures

#### Daily Moderate and Vigorous Physical Activity and Step Counts

Using triaxial accelerometer devices (ActiGraph GT3X), patients’ physical activity was objectively measured for seven consecutive days following the baseline questionnaire. In accordance with prior research, a valid monitoring day was defined as a minimum of 10-h accelerometer wear time per day [18]. To ensure reliability of accelerometer measures, solely patients with at least four valid days of accelerometer monitoring were included for further statistical analyses [19]. Using the ActiLife software, activity counts (i.e., the numbers of accelerations across 60 s) were summed and proportionally weighted to the magnitude of measured acceleration. Then, accelerometer output was categorized into MVPA by adding minutes that included 2690 activity counts or more. Minutes spent in MVPA per day were computed by averaging across valid monitoring days. Daily steps were measured for each person independent of their physical activity intensity. To adjust for univariate outliers, daily MVPA and steps levels of *z* > |3.29| were winsorized in one case to one unit lower than the next lowest value in the distribution [20].

#### Willpower Beliefs

Willpower beliefs were assessed with a 12-item scale comprising three subscales with four items, respectively [9, 21]. The measure was previously validated by Job et al. [21]. The three subscales reflected self-control domains which were assessed by using items such as “Your mental stamina fuels itself, even after strenuous mental exertion you can continue doing more of it” (strenuous mental activity domain), “After engaging in a strenuous physical task, your energy resource is usually depleted, and you must rest to get it refueled again” (strenuous physical activity domain), and “After you have resisted temptations your capacity to face upcoming temptations is still the same” (resisting temptation domain). Responses were given on a 6-point scale from “does not apply at all” (1) to “applies exactly” (6). Items referring to beliefs in limited willpower were recoded, and then, all items were averaged to one measure of willpower beliefs. Higher scores reflect a stronger agreement with beliefs in nonlimited willpower in the present study. Cronbach’s alpha for the 12 willpower beliefs items was .81.

#### Severity of the Osteoarthritis of the Knee

OAK severity was measured with the Western Ontario and McMaster Universities Osteoarthritis Index (WOMAC; [22]). Patients responded to 24 items on an 11-point scale ranging from “no symptoms” (0) to “extreme symptoms” (10). Three subscales specified symptomatology regarding (1) pain (e.g., “Please enter the amount of pain you have experienced in the past 48 hours while walking on a flat surface”), (2) stiffness (e.g., “How severe is your stiffness after first awakening in the morning?”), and (3) functional limitations (e.g., “What degree of difficulty do you have with putting on socks/stockings?”). A total WOMAC score was calculated by summarizing all three subscales. The 24 WOMAC items (range 0–240) yielded a Cronbach’s alpha of .96.

#### Body Mass Index

Based on objective measures of weight and height, patients’ BMI was computed. Standard categories for BMI include normal weight (18.5–24.9 kg/m^2^), overweight (25.0–29.9 kg/m^2^), and obesity (> 30 kg/m^2^).

#### Depressive Symptoms

Participants completed the Center for Epidemiological Studies Depression scale (CES-D; [23]). The item stem “Within the last seven days…” was followed by 20 statements concerning depressive symptoms (e.g., “I was depressed” or “I was sad”) that were rated on a 4-point Likert scale ranging from “*rarely or not at all* (less than one day)” to “*most of the time* (5–7 days).” All items were averaged to one total score. A CES-D score below 16 indicated no clinically significant symptoms, whereas a score of at least 16 indicated (sub-)clinically significant depressive symptoms (range 0–60). Cronbach’s alpha across the 20 items was .85.

### Data Analysis

Using IBM SPSS 25, Pearson correlations and descriptive statistics of study variables were calculated. Multiple regression models were run to analyze main effects of willpower beliefs on daily MVPA and steps. The SPSS PROCESS macro [24] was used to examine differential associations between willpower beliefs and physical activity indicators (daily MVPA or steps as outcomes) as a function of health demands (OAK severity, BMI, or depressive symptoms as moderators in separate models). Simple effects of sex, age, and BMI [25, 26] were accounted as additional covariates when OAK severity or depressive symptoms served as moderators. When BMI served as a moderator, in addition to sex and age, a BMI simple effect was also controlled as part of testing moderation. Unless dichotomous, all predictors were grand-mean centered. When evidence (including *p* < .10) of a willpower beliefs × hypothesized moderator interaction emerged, this was followed up by simple slope analyses and simple slope plots for moderators at one standard deviation below and above their mean. Following recommendations for moderation analyses in field studies, the range of confidence intervals has not been adjusted for multiple testing [27, 28] and results are reported as point estimates at 90% confidence intervals [29]. Nevertheless, due to potentially insufficient statistical power for testing moderations, the intervals should not be used to infer definite conclusions on the links examined.

## Results

### Descriptive Results

Accelerometer data of *n* = 238 (95.8% of the total sample) patients who had worn the accelerometer for at least four valid days were included in the present analyses. On average, patients spent 43.10 min (*SD*_mvpa_ = 27.93) per day in MVPA and walked 6301 steps per day (*SD*_steps_ = 2850). The mean OAK severity score was 73.55 (*SD*_WOMAC_ = 36.98), which is comparable or lower than reported by Woolacott et al. in a systematic review [30]. Patients’ mean BMI was 28.55 (*SD*_BMI_ = 4.87) with the majority of them being overweight or obese (*n*_overweight_ = 98, 40.5%; *n*_obese_ = 79, 32.5%). A small group of patients showed levels of depressive symptoms meeting or exceeding the cutoff for clinical significance (*n* = 41, 16.9%).

Table 1 displays descriptive statistics as well as zero-order correlations among study variables. Daily MVPA and steps were positively correlated (*r* = .76).

**Table 1:** Descriptive statistics among study variables

Variables	*M* (SD)	Min	Max	*N*	1	2	3	4	5	6	7	8
1 Daily MVPA	43.0 (27.93)	0.71	132.15	237	-	.76**	− .16*	− .04	− .18**	− .05	− .22**	.07
2 Daily steps	6301 (2850)	4.71	15.39	230		-	.18*	− .15*	− .31**	− .08	− .18**	.04
3 Willpower beliefs	3.68 (0.72)	1.25	5.42	241			-	− .05	− .15**	− .28**	.06	.06
4 OAK symptoms	73.35(36.98)	8.0	181.00	241				-	.30**	.18**	− .09	− .10
5 BMI	28.55 (4.87)	19.16	45.79	242					-	.18**	− .03	− .05
6 Depressive symptoms	10.12 (7.13)	0.0	35.0	242						-	.02	− .20**
7 Age	65.47 (7.70)	44.0	80.0	241							-	− .05
8 Gender	-	0	1	242								-

*p < .05

**p < .01

Higher scores reflect beliefs in nonlimited willpower, greater OAK severity, more depressive symptoms, and male gender

### Associations of Willpower Beliefs with Indicators of Physical Activity

In accordance with hypothesis 1, findings from multiple regression models indicated that patients who reported higher beliefs in nonlimited willpower were more likely to show higher levels of daily MVPA (*b*(*SE*) = 5.55 (2.43), *p* = .023, 95% CI (0.76, 10.35)) and daily steps (*b*(*SE*) = 559.90 (245.62), *p* = .024; 95% CI (75.88, 1043.93)).

### Differential Associations of Willpower Beliefs with Indicators of Physical Activity

Relationships between willpower beliefs and physical activity indicators were further examined as a function of patients’ health demands (their OAK severity, BMI, or depressive symptoms). Results of moderation models are displayed in Table 2.

**Table 2:** Moderator models predicting daily moderate-to-vigorous physical activity (MVPA) and steps

Moderators	OAK severity	BMI	Depressive symptoms
Outcome	Daily MVPA		
	*b* (*SE*) *p*	*b* (*SE*) *p*	*b* (*SE*) *p*
Intercept	41.85 (2.25) < .001	41.40 (2.28) < .001	4.29 (1.81) < .001
Age	− 0.81 (.23) < .001	− 0.80 (.23) < .001	− 0.84 (0.23) < .001
Gender	2.46 (3.569) .489	2.10 (3.57) .402	2.94 (3.63) .419
BMI	− 1.01 (.38) .009		− 0.99 (0.38) .008
WB	4.95 (2.43) .044	5.15 (.52) .035	5.12 (2.58) .049
Moderator	0.00 (.05) .960	− 1.02 (.36) .006	0.06 (0.27) .836
WB × Mod	− 0.14 (.07) .038	− 0.94 (.53) .074	− 0.48 (0.34) .166
∆*R*^2^	0.017	0.012	0.008
*R*^*2*^	0.123	0.119	0.116
Outcome	Daily steps		
Intercept	6270.87 (227.53) < .001	6210.04 (230.51) < .001	6085.30 (231.34) < .001
Age	− 71.06 (22.71) .002	− 66.48 (22.68) .004	− 67.27 (22.17) .003
Gender	38.42 (359.23) .915	115.70 (359.81) .748	110.47 (359.88) .792
BMI	− 159.573 (38.15) < .001		− 177.45 (36.34) < .001
WB	527.94 (247.08) .034	521.61 (245.97) .035	398.55 (255.88) .120
Moderator	− 6.16 (5.13) .034	− 175.98 (36.55) < .001	− 11.54 (26.72) .666
WB × Mod	− 8.52 (6.80) .212	− 83.56 (52.61) .114	− 110.15 (33.89) .001
∆*R*^2^	0.006	0.010	0.038
*R*^2^	0.161	0.158	0.188

*WB*, willpower beliefs; *∆R*^*2*^, variance explained by the interaction term above and beyond the other variables in the model

**Fig. 1:**
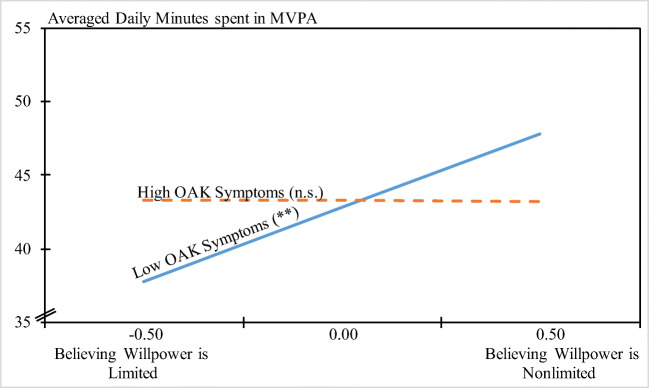
Relationship between willpower beliefs and minutes spent in moderate-to- vigorous physical activity (MVPA) for low and high levels of OAK severity. ^†^*p* < .10. *p < .05. **p < .01

OAK severity significantly moderated the willpower belief—MVPA association. Contrary to hypothesis 2, however, simple slope analyses indicated that believing in nonlimited willpower was significantly related to more MVPA for patients with lower OAK severity (*b*(SE) = 10.04 (3.24), *p* = .002, 95% CI (3.66, 16.41)). For patients with higher OAK severity, willpower beliefs and MVPA were unrelated (*b*(SE) = − 0.14 (3.65), *p* = .969 95% CI (− 7.33, 7.05); see Fig. 1).

**Fig. 2:**
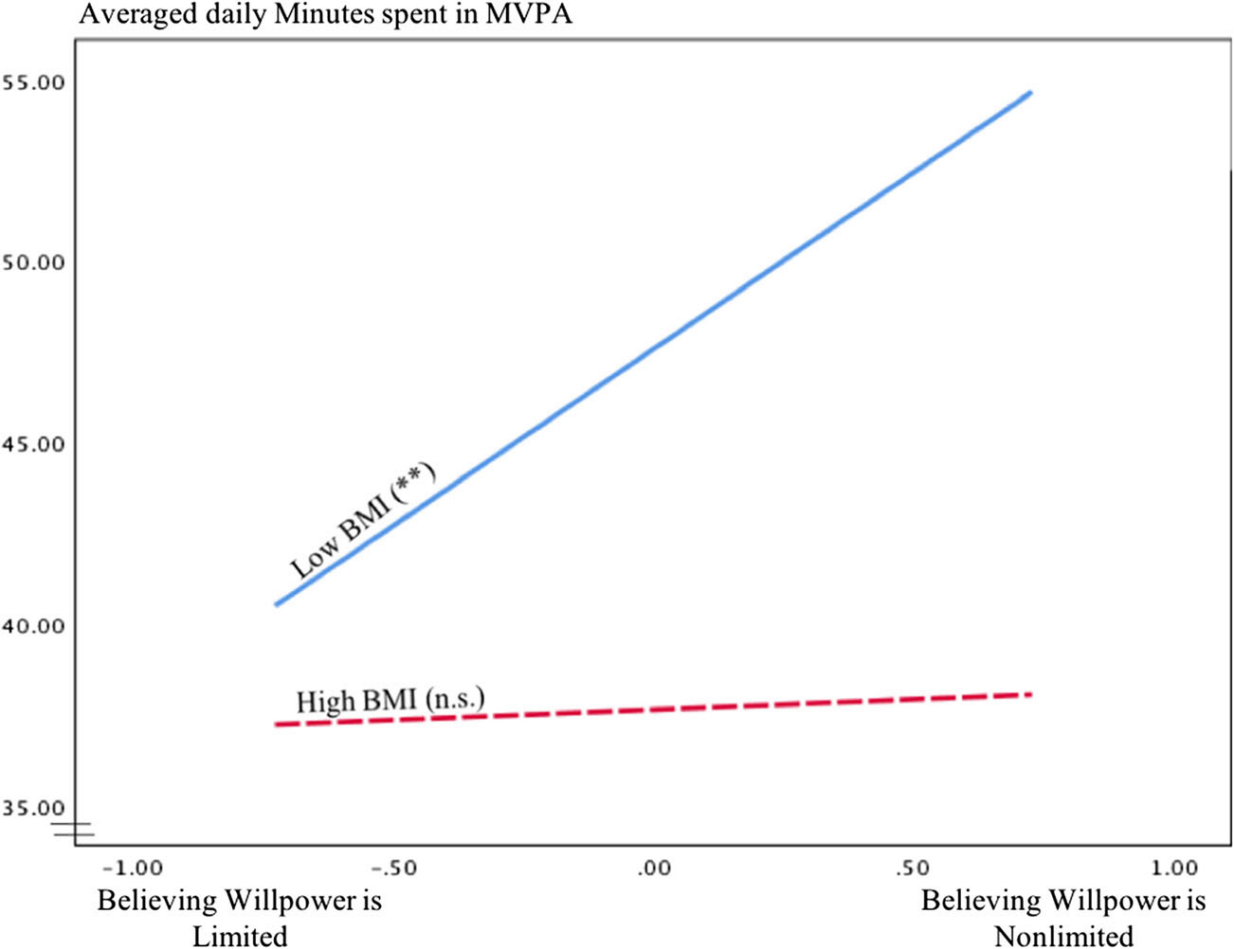
Relationship between willpower beliefs and minutes spent in moderate-to- vigorous physical activity (MVPA) for low and high levels of BMI. ^†^*p* < .10. *p < .05. **p < .01

Furthermore, a marginally significant interaction effect of BMI and willpower beliefs on MVPA (at *p* = .074) was observed. Again, in contrast to hypothesis 2, simple slope analyses showed that believing in nonlimited willpower was significantly related to more MVPA for patients with a lower BMI (low BMI mean 23.63; *b*(SE) = 9.74 (3.36), *p* = .004, 95% CI (3.11, 16.37)). For patients with a higher BMI (high BMI mean 33.43), willpower beliefs were not related with MVPA (*b*(SE) = 0.57 (3.69), *p* = .878 95% CI (− 6.70, 7.83), see Fig. 2). Depressive symptoms did not moderate willpower belief—MVPA associations.

**Fig. 3:**
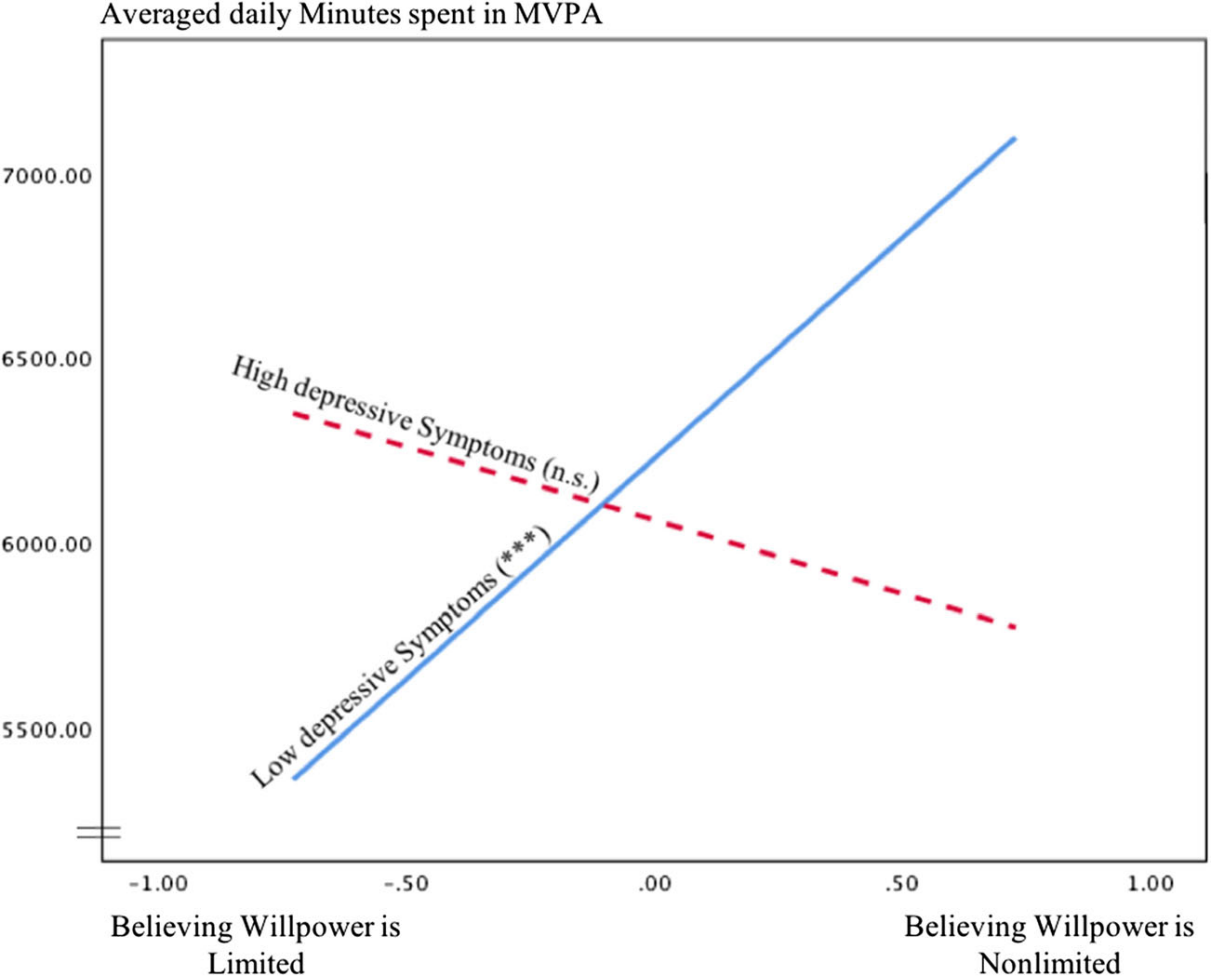
Relationship between willpower beliefs and daily steps for low and high levels of depressive symptoms. ^†^*p* < .10. *p < .05. **p < .01. ***p < .001

Relationships between willpower beliefs and daily steps were not moderated by OAK severity or BMI; however, depressive symptoms were a significant moderator. Not in line with hypothesis 2, simple slope analyses indicated that believing in nonlimited willpower was significantly linked to more daily steps in patients with lower levels of depressive symptoms (*b*(SE) = − 1195.75 (311.82), *p* < .001, 95% CI (581.22, 1810.28)). In patients with higher levels of depressive symptoms, willpower beliefs and daily steps were unrelated (*b*(*SE*) = − 398.64 (392.50), *p* = .311, 95% CI (− 1172.15, 374.88); see Fig. 3).

## Discussion

This study examined whether believing in nonlimited willpower would relate to more daily physical activity and whether this link would be more pronounced (i.e., yielding higher levels of physical activity) in patients with OAK who face relatively higher health demands (i.e., demands 1 *SD* above the sample average). In accordance with hypothesis 1, believing that willpower is nonlimited was associated with more objectively assessed physical activity (i.e., daily MVPA and steps). Contradicting hypothesis 2, for patients facing OAK severity, BMI, or depressive symptoms 1 *SD* above the sample average, willpower beliefs were unrelated with daily MVPA or steps. However, interactions between willpower beliefs and OAK symptoms as well as BMI (as a statistical trend) were linked to MVPA, whereas depressive symptoms did not further qualify the willpower belief—MVPA link. Simple slope analyses revealed that nonlimited willpower beliefs combined with below-average OAK severity as well as with below-average BMI (i.e., 1 *SD* below mean) were associated with more physical activity. Moreover, the relationship between willpower beliefs and average daily steps was particularly high for patients who reported depressive symptoms below average, whereas OAK severity and BMI did not moderate the relationship between willpower beliefs and steps. The additional variances explained by the willpower belief × health demand interactions were small (i.e., 1–3%), which is typical for interactions found in field studies [31, 32], but is nevertheless considered meaningful [33].

The general positive relationship between willpower beliefs and daily physical activity suggests that believing in nonlimited willpower promotes successful self-control in patients with OAK. This is in line with prior research that showed individuals who believed in nonlimited willpower to exhibit superior everyday self-regulation [9, 11, 12, 14]. However, the present findings also suggest that willpower beliefs are unrelated to physical activity given more severe (i.e., above average) health demands, contrasting recent work suggesting that willpower beliefs enable self-control especially under challenging conditions [9, 11–14].

Yet, except for one study examining patients with type 2 diabetes [9], previous research on willpower beliefs has primarily investigated healthy populations [11–16].

### Theoretical Implications

The current study provides some evidence that positive effects of a nonlimited willpower belief might be restricted. Chronic conditions, especially for those with severe symptoms, might pose boundary conditions under which self-regulation is no longer predicted by people’s beliefs about willpower. Evidence of a moderation of the willpower belief—physical activity link by health demands—are in line with Vohs and colleagues’ [34] suggestion that under conditions of more severe demands, willpower beliefs might no longer be beneficial.

The theoretically interesting question following from the present results is what processes may account for the lack of an effect of willpower beliefs in participants who reported demands that were above the sample’s average. Research on mechanisms of willpower beliefs suggested two routes explaining the negative effect of a limited willpower theory on self-control performance: changes in self-efficacy and motivation. Exerting self-control on one occasion has been shown to temporarily reduce self-efficacy in people with a limited willpower theory and, on the other hand, triggers a motivational shift towards rest and relaxation [15, 16]. In accordance with social cognitive theory [35, 36], this previous research would suggest that having higher efficacy should boost individuals’ self-regulatory skills in the face of challenging circumstances. In the following, we will elaborate how more severe demands (i.e., above average) might disrupt the unfolding of these two processes.

First, when demands are high, failures in self-control might be more likely on initial self-regulatory attempts. This could reduce self-efficacy even in those believing in nonlimited willpower. After all, a nonlimited willpower belief concerns the perceived ability to keep one’s self-control performance on a high level over time. It does not concern one’s overall self-control ability. Accordingly, initial failures to exert self-control might undermine any possible positive effects of a nonlimited theory on self-efficacy and subsequent self-control. For example, a patient suffering from severe OAK symptoms might feel incapable of walking to the bus station in the morning and takes the car instead. Such a low level of self-efficacy and self-control performance has not been caused by thoughts about the availability of self-control resources but, instead, directly follows from the severity of experienced impairments. A reverse causation is also possible, namely when self-efficacy is high in the first place, people are inclined to invest more effort and persistence in light of challenging demands, whether or not their willpower beliefs are limited or nonlimited.

Second, it is possible that high health demands reduce the motivation to engage in compensatory efforts in all patients, limited as well as nonlimited theorists, due to a relatively low return of investment experience. If the engagement in physical activity is highly painful, the possible resulting benefits might not outweigh the costs of exercising. Patients might thus accommodate the standards and goals regarding physical activity. Previous research suggests that such a reduction in compensatory efforts in people experiencing heavy health declines might actually buffer the perception of losses and deficits [37].

In sum, the present research provides some evidence that the positive effects of willpower beliefs might reach their limits when people face chronic high health demands. These results, however, have to be treated cautiously, since the pattern has not been predicted and the statistical power may have been insufficient to draw strong conclusions about a nonsignificant association [38]. Future research should be dedicated to the investigation of mechanisms explaining self-control engagement and success as well as its functionality under conditions of high chronic demands.

### Differential Effects

Although daily MVPA and steps showed a high empirical overlap, distinct moderator effects were found in models with MVPA or steps as outcomes. Whereas MVPA reflects only upper ranges of intensity in physical activity (i.e., at least moderately strenuous activities), daily steps may also include light activity, such as walking at a slow pace, which generates varying ranges of task difficulty in outcomes. Moreover, positive correlations of depressive symptoms with OAK severity and BMI indicated an overall higher level of health demands in patients with more depressive symptoms or likewise an overall lower level of health demands in patients with lower depressive symptoms. This might explain the more distinct moderating function of depressive symptoms concerning willpower belief associations with daily steps—or outcomes that included a wider range of task difficulty.

Strengths of this study included the objective assessment and use of two facets of physical activity representing varying ranges of physical activity intensity. Moreover, prior research mainly examined willpower beliefs in the context of psychological self-control demands (i.e., workload, academic stress, laboratory self-control tasks). In the present study, willpower beliefs were investigated in the context of changing one’s physical activity that requires overcoming physical demands.

### Limitations

However, conclusions must be drawn with caution as the present findings are tempered by some limitations. First, definite conclusions cannot be drawn from the results, as statistical analyses were potentially insufficiently powered to detect moderation processes. Second, compared with findings from prior studies on OAK patients, the present sample on average generated relatively high levels of MVPA [3], which may limit generalizability of the present results to the population of OAK patients and may also have contributed to the underestimation of effects. Moreover, implied predictive direction remains inconclusive, as most of the variables were measured at one point in time although the dependent variables (physical activity indicators) were assessed during the week following baseline self-reports. For instance, it is also conceivable that depressive symptoms contribute to beliefs in limited willpower and counteract the engagement in more frequent daily physical activity. Additionally, future research should directly test the idea that a nonlimited willpower theory is functional under somewhat high (manageable) demands but no longer functional under extremely high (non-manageable) demands. A critical question in this context is how to define and quantify at what level demands get extreme and non-manageable. There is some risk for circularity, when levels of demands, at which willpower theories are no longer predictive, are defined as high or even severe. Moreover, severity of a specific demand might also depend on other characteristics of a given population (e.g., whether multiple demands come together and what resources to cope are available). Future research needs to specify a priori what different levels of demands mean and what effects are expected with regard to willpower theories as well as possible downstream indicators of successful coping and adjustment. Also, future studies targeting willpower beliefs to promote physical activity in the face of chronically high health demands are warranted to encourage replication with adequately powered analyses.

In conclusion, this study provided additional insight into the interplay between willpower beliefs and varying health demands when it comes to the performance of resource-demanding health behaviors in adults burdened with chronic disease. Whereas beliefs in nonlimited willpower were generally related with higher physical activity in OAK patients; excessive demands produced by OAK symptom severity, BMI, and depressive symptoms appeared to counteract this effect. The present study thus contributes evidence on possible boundary conditions of the positive effects of a nonlimited theory about willpower with regard to actual health behavior. Even though it is overall beneficial to believe that self-control capacity is rather not limited, the belief in nonlimited willpower might not be the solution for people who struggle with more severe chronic demands.
